# Enhancing phytochemical composition and nutritional profiles in dry bean varieties through roasting

**DOI:** 10.1002/jsfa.70106

**Published:** 2025-08-12

**Authors:** Olumide O Fashakin, Karen Cichy, Ilce G Medina‐Meza

**Affiliations:** ^1^ Department of Biosystems and Agricultural Engineering Michigan State University East Lansing Michigan USA; ^2^ USDA‐ARS, Sugarbeet and Bean Research Unit East Lansing Michigan USA

**Keywords:** *Phaseolus vulgaris* L., common beans, phytochemicals, lipid profile, antioxidants, roasting

## Abstract

**BACKGROUND:**

Dry beans and chickpeas are nutritionally valuable legumes widely consumed worldwide. This study examines the impact of oven roasting (110 °C for 70 min) on the phytochemical, antioxidant, fatty acid and saponin profiles of 12 pulses, including 11 common beans and 1 chickpea.

**RESULTS:**

Roasting significantly affected the phytochemical composition and antinutritional factors with distinct variations among samples. Cranberry bean CRAN‐1 exhibited the highest total phenolic content in raw samples (19.12 g kg^−1^ GAE g^−1^ DW), which decreased by 6.79% post‐roasting. Pigmented beans including dark red kidney and CRAN‐2 showed strong antioxidant activities pre‐roasting (DPPH scavenging: 73.94% and 50.16%, respectively). CRAN‐2 also had the highest anthocyanin content (0.057 to 0.036 g kg^−1^ DW) among pigmented cultivars, which decreased slightly after roasting. Non‐extractable polymeric proanthocyanins remained stable, while saponin content significantly decreased (up to 77% in some cultivars), suggesting improved flavor. Stigmasterol and *β*‐sitosterol were the predominant phytosterols with enhanced availability after roasting. Fatty acid analysis revealed that saturated fatty acids are predominant, ranging from 54.7% (Manteca) to 70% (Great Northern), while the fatty acid composition was similar across all cultivars.

**CONCLUSION:**

These findings underscore that roasting under mild controlled conditions retains and improves key bioactive compounds in common bean flours. Pigmented varieties, in particular, offer superior antioxidant and phytochemical properties. This research provides a foundation for incorporating roasted pulses into functional formulations with both dietary and industrial applications. © 2025 The Author(s). *Journal of the Science of Food and Agriculture* published by John Wiley & Sons Ltd on behalf of Society of Chemical Industry.

## INTRODUCTION

Common beans are some of the most important legumes valued for their nutritional content, including dietary fiber (20–31%), lipids (1–3%) and proteins (16–33%), comprising globulins (54–79%) and albumins (12–30%). They contain functional phytochemicals such as phenolic acids, flavonoids, proanthocyanins, vitamins, minerals and antioxidants.^1^, ^2^, ^3^ Despite their nutritional and health benefits, bean consumption in the USA is below the recommended dietary intake of 1–3 cups per week.^4^, ^5^ According to the US Department of Health and Human Services and the US Department of Agriculture,^6^ the typical US diet lacks essential nutrients, including dietary fiber, iron, potassium, magnesium and health‐beneficial phytochemicals with high antioxidant activity, all abundant in beans.^7^

Bean consumption is associated with reduced risk of obesity, diabetes and cardiovascular diseases, which are prevalent among all age groups in the USA.^8^ Increasing bean consumption aligns with public health strategies to ameliorate cardiovascular and chronic diseases.^6^ The chemical composition of common beans differs due to genotype, environmental conditions, storage and processing conditions, all playing an important role in the phytochemical profile and antinutritional factors.^9^, ^10^, ^11^

Traditionally, common beans are consumed as whole boiled pulses, and there is growing interest for innovative value‐added applications, such as their conversion into flour.^12^ Processing methods like roasting, a dry‐heat technique, can reduce antinutritional components (e.g. tannins, phytates and lectins), enhance flavor and improve the sensory and functional properties of bean flour.^13^, ^14^, ^15^ Given the nutritional density of beans, the development of bean flour can be used in functional food products that meet consumer demands for convenience and address dietary health and nutrition needs.^16^

To enhance the consumer acceptance and health‐promoting and nutritional attributes of bean flours, pre‐milling processing methods such as roasting have been employed. Roasting is a thermal processing technology that improves the sensory appeal of beans, influences their chemical composition and significantly alters phytochemical profiles by increasing antioxidant bioavailability and reducing antinutrients that interfere with nutrient absorption.^17^

Understanding the effects of roasting across different bean varieties is essential to optimizing the health‐promoting and nutritional quality of bean‐derived products. Pigmented beans are of particular interest due to the presence of anthocyanins and other polyphenols, with known antioxidant properties. When incorporated as flour into everyday foods such as pasta, bread and baked goods, beans boost the nutritional profile while maintaining desirable organoleptic properties.^16^ Characterization of the lipid profile and phytosterols in roasted beans may provide additional insights into their role in supporting cardiovascular health. The study reported here aimed to evaluate the impact of roasting on key bioactive components, including phytochemicals, antioxidants, phytosterols, lipid composition and saponins, in 11 bean varieties and one chickpea variety, assessing their potential as a functional food ingredient for the development of value‐added products.

## MATERIALS AND METHODS

### Chemicals

Standards of campesterol, stigmasterol, squalene, *β*‐sitosterol, oleanolic acid, gallic acid and procyanidin B were purchased from Cayman (Cayman Chemical, Ann Arbor, MI). Supelco 37 FAME mix, tridecanoid acid, 5*α*‐cholestane and pyridine were obtained from Sigma‐Aldrich. HPLC‐grade methanol, ethanol and *n*‐hexane were purchased from VWR Chemicals BDH (Radnor, PA). HPLC‐grade methanol, ethanol,1‐butanol and potassium chloride (KCl) were from JT Baker (Allentown, PA) and diethyl ether was purchased from Fisher Chemical (Pittsburgh, PA). Sodium sulfate anhydrous (Na_2_SO_4_) and sodium chloride were also purchased from VWR BDH Chemicals.

### Pulse samples

**Figure 1:**
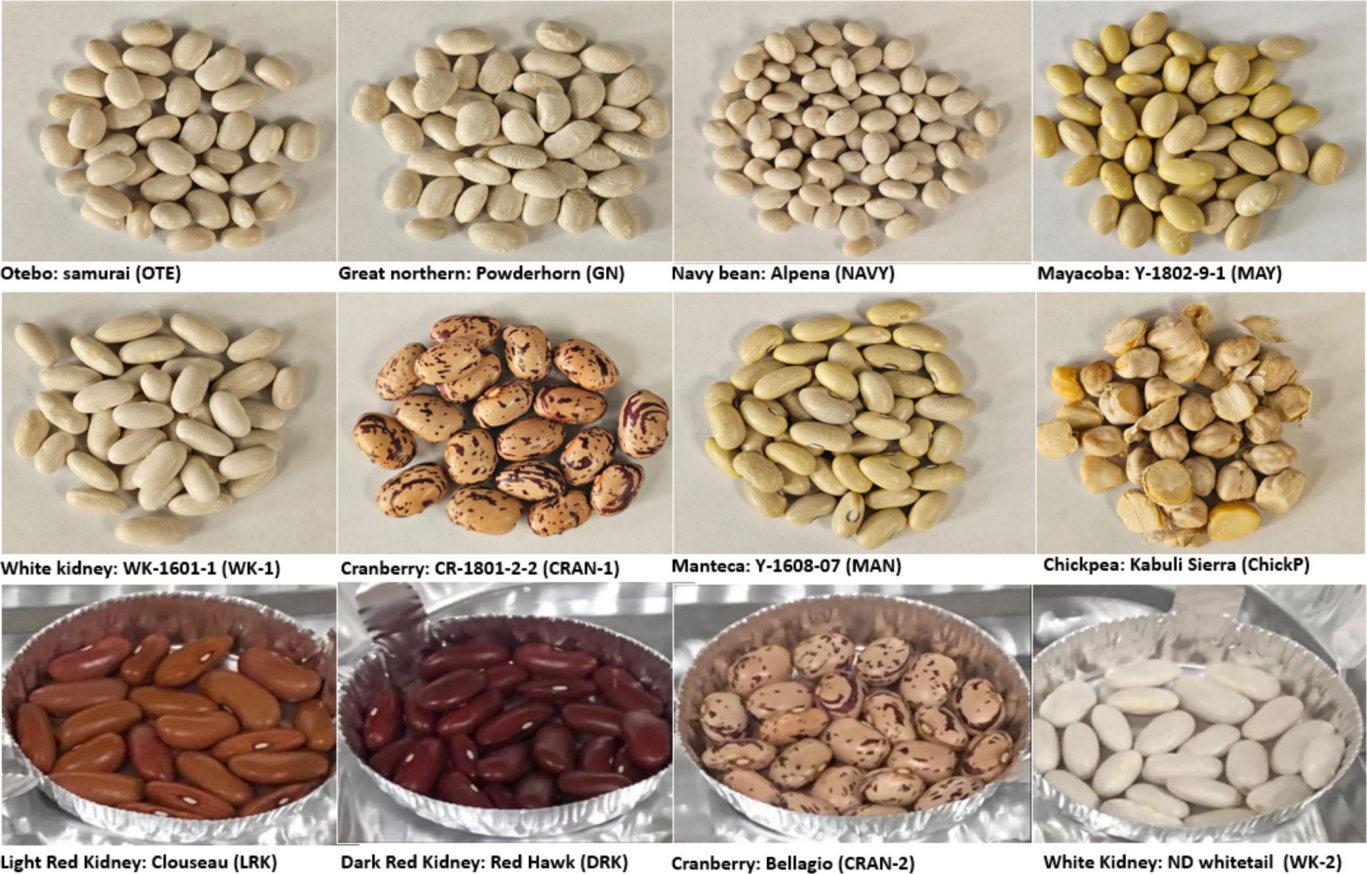
Photos of 11 newly bred bean (*Phaseolus vulgaris* L.) varieties and chickpea (*Cicer arietinum*) analyzed (Otebo: Samurai (OTE); Great Northern: Powderhorn (GN); Navy Alpena (NAVY); Mayacoba: Y1802‐9‐1 (MAY); White Kidney: WK1601‐1 (WK‐1); Cranberry: CR1801‐2‐2 (CRAN‐1); Manteca: Y1608‐07 (MAN); Chickpea: Sierra (ChickP); Red Hawk (DRK); Bellagio (CRAN‐2); Clouseau (LRK); ND Whitetail (WK‐2)).

Twelve pulse genotypes (seven commercial cultivars and four USDA‐ARS advanced breeding lines) and one Kabuli chickpea were evaluated in this study (Fig. 1). Beans were grown in 2022 at the Michigan State University, Montcalm Research Farm in Entrican, MI in a replicated trial of three replicates per entry planted in a randomized complete block design. Each plot was four 6.1 m long rows with 0.51 m rows with approximately 13 seeds planted per meter. The center two rows were the experimental genotype, and the outer two rows were a uniform border. The field trial was managed following standard agronomic practices and harvested with a Hege 150 plot combine. The chickpea was grown by a commercial farm in Montana in 2022 and donated by a pulse processing company. Following harvest, beans were cleaned to remove debris and damaged seeds and were stored at room temperature. An amount of 100 g of whole beans was rinsed with distilled water (1:3 w/v) until all dust had been successfully removed and allowed to dry overnight on a paper towel‐lined sheet tray, after which the seeds were further sorted to remove any remaining debris. Cleaned beans were roasted at 110 °C for 70 min using a convection oven (Fisher Scientific Isotemp Gravity Oven, 100 L) and allowed to cool for 4 h at room temperature. Both raw and roasted samples were milled using a 0.5 mm mesh size Hammer mill (Polymix® Laboratory Grinding Mills, PX‐MFC 90 D, Kinematica). The bean flour was packed in a sealed bag and stored at 4 °C to minimize volatile loss until needed for further analysis. A description of the varieties is presented in Table 1.

**Table 1:** Common bean germplasm used in this study

Sample market class and cultivar name	Seed coat color	Raw sample abbreviation	Roasted sample abbreviation
Otebo: Samurai	White	OTE	OTE‐R
Great Northern: Powderhorn	White	GN	GN‐R
Navy: Alpena	White	NAVY	NAVY‐R
White Kidney: WK1601‐1	White	WK‐1	WK‐1‐R
White Kidney: ND Whitetail	White	WK‐2	WK‐2‐R
Manteca: Y1608‐07	Pale yellow	MAN	MAN‐R
Mayacoba: Y1802‐9‐1	Yellow	MAY	MAY‐R
Cranberry: CR1801‐2‐2	Tan/pink mottled	CRAN‐1	CRAN‐1‐R
Cranberry: Bellagio	Tan/pink mottled	CRAN‐2	CRAN‐2‐R
Light Red Kidney: Clouseau	Light red	LRK	LRK‐R
Dark Red Kidney: Red Hawk	Dark red	DRK	DRK‐R
Chickpea: Sierra	Yellow	ChickP	ChickP‐R

### Extraction of phenolics from flour

Bean flour (100 mg) was placed into a 15 mL centrifuge tube, and 5 mL of 80:20 (v/v) methanol–water was added. The mixture was vortexed vigorously for 1 min and then sonicated in an ultrasonic water bath for 10 min. The sample was subsequently centrifuged at 2057 × *g* for 10 min at 4 °C. The extraction was repeated twice, and the resulting supernatants were pooled into a new tube. The combined extract was kept at −20 °C for further use, while the residual pellets were retained for non‐extractable polymeric proanthocyanidin (NEPA) analysis.

#### Quantification of total phenolic content

Total phenol quantification was performed using the Folin–Ciocalteu method with slight modifications.^18^ Briefly, 100 μL of Folin–Ciocalteu reagent was added to an aliquot of 100 μL of crude extracts in an Eppendorf® tube, the mixture was vortexed and then allowed to stand for 2 min. An amount of 800 μL of Na_2_CO_3_ solution (5% w/v) was added to the mixture, vortexed and left for 30 min at room temperature. The absorbance was measured at 765 nm. The measurement was conducted using a double‐beam UV‐6300PC spectrophotometer (VWR, Radnor, PA). Results were expressed as equivalents of gallic acid/g kg^−1^ DW.

#### Determination of total flavonoids

Total flavonoids were determined spectrophotometrically as previously reported by Chen *et al*.^18^ Briefly, 435 μL of the aliquot from the methanol extract was mixed with 435 μL of 2% AlCl_3_ and 645 μL of 0.6 mol L^−1^ sodium carbonate. The mixture was allowed to stand for 150 min at 20 °C, then the absorbance was read at a wavelength of 440 nm. Total flavonoids were expressed as g kg^−1^ DW ± standard deviation.

#### FRAP and DPPH assays

The ferric‐reducing antioxidant power (FRAP) and 2,2‐diphenyl‐1‐picrylhydrazyl (DPPH) assays were conducted according to our previous studies.^18^, ^19^ Samples were incubated at 37 °C for 4 min. Then absorbance was measured at 593 nm against a blank of FRAP solution incubated at 37 °C. Results were expressed as mg g^−1^ ferrous sulfate eq. DW. For DPPH, 100 μL of methanolic extract was mixed with 1400 μL of DPPH solution, vortexed and ultrasonicated for 1 min. Subsequently, the sample was incubated at room temperature for 30 min, after which the absorbance was measured at 515 nm using a UV–visible double‐beam Shimadzu spectrophotometer (UV‐6300PC, VWR, Radnor, PA). Methanol was used as a blank, and results were described as percentage of radical scavenging activity.

DPPH scavenging activity was calculated as described in Eqn (1):

(1) $$\text{DPPH radical scavenging activity}\;\left(\%\right)=\frac{A_{\text{control}}-A_{\text{sample}}}{A_{\text{control}}}\times 100$$

where *A* (control and sample) are the optical densities at 517 nm. The results were described as percentage of antioxidant capacity.

### Total carotenoids

The carotenoid content was estimated according to Zia *et al*.^20^ Briefly, 200 mg of bean flour was extracted for carotenoid extraction with 2 mL of acetone. Then, the mixture was centrifuged for 5 min at 2057 × *g* at 4 °C, after which the upper phase was recovered in a new tube. The absorbance of the carotenoids was measured using a glass cuvette at a wavelength of 470 nm using a UV–visible double‐beam Shimadzu spectrophotometer (UV‐6300PC, VWR, Radnor, PA). Acetone was used as blank, and results were expressed as g kg^−1^ *β*‐carotene DW.

### Total monomeric anthocyanin content

The total monomeric anthocyanin content was determined by the pH differential method.^21^ Absorbances at pH 1.0 and 4.5 were determined at 520 and 700 nm using a UV–visible spectrophotometer, with distilled water as the blank. Results are expressed as g kg^−1^ DW cyanidin‐3‐glucoside eq.

### Total proanthocyanidin content by vanillin assay

Proanthocyanidins were determined following the vanillin assay method.^22^ Briefly, 500 μL of extract was mixed with 1.25 mL of vanillin solution (1% w/v) and 1.25 mL of H_2_SO_4_ solution (25% w/v). The mixture was vortexed and allowed to stand at room temperature for 15 min, protected from light. The absorbance was read using a glass cuvette at 500 nm in a UV–visible spectrophotometer (VWR, Radnor, PA). Blanks were prepared simultaneously as all reagents without sample and results were expressed as g kg^−1^ procyanidin B1 eq. DW.

### Non‐extractable polymeric proanthocyanidins

NEPAs were quantified according to a previously reported method.^23^ The pellet remaining from phenolics extraction was dried using a nitrogen stream. Then 3 mL of reagent containing 0.015 g of FeCl_3_ in 25 mL of HCl–butanol (5:95 v/v) was added and vortexed vigorously for 1 min. The mixture was heated at 80 °C for 90 min, allowed to cool in ice and centrifuged at 2057 × *g* for 10 min at 4 °C. Following centrifugation, the supernatant was recovered. This step was repeated a second time with 2 mL of reagent without heating. A total of 5 mL was recovered from both supernatants, vortexed and read with a UV–visible double‐beam spectrophotometer (UV‐6300PC, VWR, Radnor, PA) at 450 and 555 nm, respectively. Results were reported as g kg^−1^ procyanidin B2 DW.

### Fat extraction

An amount of 1 g of flour was placed in a tube, and 10 mL of *n*‐hexane was added, capped and vortexed thoroughly. The mixture was sonicated in a VWR® ultrasonic cleaner bath containing iced water for 10 min, centrifuged at 804 × *g* for 10 min at 4 °C and then the supernatant was recovered. The extraction was repeated a second time using 5 mL of *n*‐hexane. Then the solvent was dried under a nitrogen stream, and the fat was recovered. An amount of 5 mL of hexane–isopropanol (4:1 v/v) was used to preserve the fat at −20 °C until further use.

### Quantification of fatty acid methyl esters

Fatty acid methyl esters were quantified according to Chen *et al*.^18^ with a few adjustments. An amount of 20 mg of oil was used, and after methylation, the hexane layer was recovered for fatty acid profiling.

### Quantification of phytosterols

Quantification of phytosterols was conducted as previously reported.^18^ An amount of 30 mg of oil was weighed, and 40 μg of the internal standard (5*α*‐cholestane) was added to the oil after drying under nitrogen gas. Then 2 mL of 50% (w/v) ethanolic KOH was added to the mixture in screw‐top tubes. Tubes were heated for 60 min at 70 °C and cooled in ice. Then, 4 mL of hexane and 1 mL of distilled water were added, centrifuged at 2057 × *g* for 10 min and the unsaponifiable fraction was collected. The extraction was repeated with 2 mL of hexane. An amount of 100 μL of the unsaponifiable fraction was derivatized by adding 100 μL of anhydrous pyridine and 150 μL of bis(trimethylsilyl)trifluoroacetamide. The mixture was heated at 70 °C for 1 h, then transferred into a GC vial. An amount of 2 μL of the derivatized sample was injected into a GCMS‐QP2010 SE Shimadzu® coupled with a capillary column (30 m × 0.25 mm × 0.25 μm). Injector and detector temperatures were both set at 280 °C, whereas the oven temperature profile was programmed from 250 to 280 °C at 3 °C min^−1^, from 280 to 300 °C at 2 °C min^−1^ and held for 5 min. A carrier pressure of 100 kPa was used, the split ratio was 50.0 and the total flow was 43.6 mL min^−1^. Identification was done by comparison of retention time with that of the corresponding pure standards and confirmed by the EI mass fragmentation.

### Sapogenin extraction and quantification of total saponin

The total saponin content was measured according to Medina‐Meza *et al*.^23^ with slight modifications. An amount of 1 g of defatted flour was sonicated with 80% methanol for 10 min and centrifuged at 2057 × *g* for 10 min to extract the crude sapogenin. A 5 mL aliquot of crude sapogenin was hydrolyzed with 2 mL of 6 N HCL, heated to 110 °C for 2 h and cooled at room temperature before centrifuging at 2057 × *g* for 5 min. The hydrolysate was neutralized with 1 mL of 6 N concentrated NH_4_OH solution. Afterward, saponin was extracted with 10 mL of ethyl acetate and filtered through Na_2_SO_4_ anhydrous bed with Whatman paper. The supernatant obtained after filtration was reduced to 2 mL under a nitrogen stream. Then, 500 μL of the extract was used for the color agent reaction and read at 527 nm with a UV–visible spectrophotometer. Results were reported in g kg^−1^ oleanolic acid equivalent DW.

### Statistical analyses

Results were expressed as mean value ± standard deviation of independent biological extractions (*n* = 3). One‐way analysis of variance (ANOVA) was used to compare the means, and a *post hoc* test was then performed by Tukey's procedure, using a *P* value of 0.05. Pearson correlation was performed at *P* ≤ 0.05, 0.01 and 0.001. IBM SPSS Statistics V28 was used, and MATLAB Version R2024b was used to produce the principal component analysis (PCA) figures.

## RESULTS AND DISCUSSION

### Determination of total phenolics, total flavonoids and antioxidant capacity in dry beans and chickpeas

Table 2 presents the total concentration of phenolics and flavonoids and antioxidant capacity (DPPH and FRAP) of raw and roasted bean flours. The total phenolic content varied widely between the raw and roasted samples. Roasting impacted the total phenolic content differently depending on the variety, such that some were reduced, some unchanged and some showed increased phenolic content compared to the raw form. Overall, CRAN‐1 (CR1801‐2‐2, Cranberry genotype) has the highest total phenolic concentration with 19.12 g kg^−1^ DW (raw) and 17.82 g kg^−1^ DW (roasted) which are higher than those of pigmented dark red kidney (DRK) beans (10.30; 11.42 g kg^−1^ DW) and CRAN‐2 (16.52; 10.06 g kg^−1^ DW) for both raw and roasted samples, respectively. The Manteca (MAN) and light red kidney (LRK) beans also show significant differences in total phenolic contents when roasted compared to the raw sample (*P* ≤ 0.05). Notably, the concentration of total phenolics was higher in pigmented bean varieties (CRAN‐1, DRK, CRAN‐2 and LRK) compared to non‐pigmented beans. This is in line with other research that reported that higher phytochemical activities are linked to bean pigmentation.^24^, ^25^ Conversely, the reduction observed in some varieties after roasting may be due to heat polymerization in phenolic compounds, leading to an increase in molecular weight and insolubility.^26^

**Table 2:** Phenolics, antioxidant activities and carotenoid content of raw and roasted beans

	Total phenolics (g kg^−1^ gallic acid equivalents DW)		Total flavonols (g kg^−1^ quercetin eq. DW)		DPPH (% of radical scavenging activity)		FRAP (g kg^−1^ ferrous sulfate eq. DW)		Carotenoid (g kg^−1^ *β*‐carotene DW)	
	Raw	Roasted	Raw	Roasted	Raw	Roasted	Raw	Roasted	Raw	Roasted
OTE	4.69 ± 0.01	5.35 ± 0.53	0.87 ± 0.15	0.73 ± 0.03	54.01 ± 1.27	55.81 ± 0.73	12.03 ± 1.00	12.64 ± 0.17	n.d.	n.d.
GN	4.46 ± 0.13	4.62 ± 0.22	4.67 ± 0.55	0.61 ± 0.07	54.48 ± 0.18	56.29 ± 0.40	13.08 ± 0.32	12.93 ± 0.14	n.d.	n.d.
NAVY	5.29 ± 0.80	5.01 ± 0.52	1.43 ± 0.03	0.76 ± 0.16	56.79 ± 0.45	57.53 ± 0.37	13.78 ± 0.58	14.54 ± 0.42	n.d.	n.d.
WK‐1	6.39 ± 0.54	5.44 ± 0.27	6.72 ± 0.40	7.55 ± 0.56	55.69 ± 0.28	54.95 ± 0.27	13.10 ± 0.10	12.37 ± 0.25	n.d.	n.d.
WK‐2	4.34 ± 0.23	4.45 ± 0.32	4.79 ± 1.04	3.73 ± 0.34	49.93 ± 1.42	52.20 ± 0.95	21.44 ± 0.19	22.33 ± 0.76	0.002 ± 0.0	0.002 ± 0.00
MAN	7.70 ± 0.35	6.89 ± 0.33	7.31 ± 0.33	5.99 ± 0.20	55.87 ± 0.77	56.14 ± 0.22	11.24 ± 0.38	12.62 ± 0.05	0.003 ± 0.0	0.003 ± 0.00
MAY	6.07 ± 0.37	6.64 ± 0.10	5.06 ± 0.30	4.16 ± 0.27	56.40 ± 0.54	56.85 ± 0.77	13.66 ± 0.22	13.93 ± 0.16	0.002 ± 0.0	0.003 ± 0.00
CRAN‐1	19.12 ± 0.33	17.82 ± 0.94	7.70 ± 0.11	7.23 ± 0.23	73.94 ± 0.67	72.97 ± 1.38	53.53 ± 1.23	45.37 ± 0.99	0.003 ± 0.0	0.003 ± 0.00
CRAN‐2	16.52 ± 1.41	10.06 ± 1.10	6.52 ± 0.30	4.47 ± 0.13	50.16 ± 1.70	30.08 ± 1.23	69.49 ± 0.48	68.00 ± 2.11	0.003 ± 0.0	0.004 ± 0.00
LRK	10.62 ± 0.77	10.45 ± 1.39	5.43 ± 0.89	4.51 ± 0.23	31.68 ± 2.38	31.88 ± 0.64	32.48 ± 2.56	30.76 ± 1.39	0.004 ± 0.00	0.004 ± 0.00
DRK	10.30 ± 1.04	11.42 ± 0.31	6.35 ± 0.52	4.18 ± 0.28	24.58 ± 2.92	32.09 ± 1.72	31.40 ± 0.76	34.85 ± 0.00	0.003 ± 0.0	0.003 ± 0.00
ChickP	5.17 ± 0.81	5.11 ± 0.41	1.60 ± 0.23	0.92 ± 0.17	50.31 ± 1.39	50.73 ± 1.59	18.31 ± 0.58	19.04 ± 0.83	0.012 ± 0.0	0.011 ± 0.00
*P* value (raw *versus* roasted)	NS		**		NS		NS		NS	

Data represent mean ± standard deviation (*n* = 3). All data are reported on a dry basis (DB). Data were analyzed by one‐way ANOVA according to an analysis of variance and Tukey's multiple mean comparison test (*P* < 0.05). ‘n.d.’ = not determined. Otebo: Samurai (OTE); Great Northern: Powderhorn (GN); Navy Alpena (NAVY); Mayacoba: Y1802‐9‐1 (MAY); White Kidney: WK1601‐1 (WK‐1); Cranberry: CR1801‐2‐2 (CRAN‐1); Manteca: Y1608‐07 (MAN); Chickpea: Sierra (ChickP); Red Hawk (DRK); Bellagio (CRAN‐2); Clouseau (LRK); ND Whitetail (WK‐2). All analyses are measured in DW (dry weight). The significance level was checked between two pairs of raw and roasted at *P ≤ 0.05; **P ≤ 0.01; ***P ≤ 0.001; and NS = non‐significant.

The highest flavonoid content in raw flour was found in CRAN‐1 with a value of 7.70 g kg^−1^. Noticeably, roasting leads to a decrease in flavonoids in all varieties except WK‐1 which shows a slight increase from 6.72 to 7.55 g kg^−1^ after roasting. The flavonoid concentration aligns with the results of Siah *et al*.,^27^ who reported reduced antioxidant and total phenolics, flavonoids and antioxidant capacity, and increased phenolic and flavonoid contents after a prolonged roasting at 150 °C for 60 min. It has been reported that roasting differentially influences the phenolic and flavonoid components of seeds.^28^ Somporn *et al*.^29^ suggested that this may be due to the different reactivities of phenolic compounds being distinctly affected by heating processes, and the difference in their chemical structures, causing variation in binding status.

The influence of roasting on the antioxidant activities of bean flour was investigated based on the change in the radical scavenging capacities (DPPH) and FRAP, as presented in Tables 2 and 4. The DPPH radical scavenging activities varied among bean varieties, with no significant differences after roasting, including in chickpea (ChickP). For example, the highest DPPH was observed in the Cranberry genotype (CRAN‐1), with a slight reduction from 73.94% to 72.97% (before and after roasting, respectively) and was better than those of other bean varieties, ranging from 24.58% to 56.40% for raw samples and 31.88–57.53% for roasted samples of other varieties. That for CRAN‐2 also reduced from 50.16% to 30.08% after roasting, whereas other varieties underwent a slight increase, ranging from a 0.48% (MAN) to a 30.45% (DRK) increase. The increase in DPPH activities in some varieties and a decrease in others indicates that roasting affects the chemical composition of the samples. Further, antioxidant activities were positively correlated with the phenolic compositions of the different varieties.^30^ Koriyama *et al*.^31^ suggested that roasting has the potential to increase the antioxidant activities of soybeans at a temperature above 190 °C. Similar to the DPPH findings, a slight decrease was also observed in the FRAP value after roasting (53.53 ± 1.23 and 45.37 ± 0.99 mg g^−1^ DW) for CRAN‐1. The lowest FRAP value was observed for Mayacoba (MAY) (with a slight increase after roasting (11.24–12.62; *P* < 0.05)). CRAN‐1 variety has the potential for higher antioxidant activity as a higher DPPH corresponds to a strong antioxidant potential, and the increase in the antioxidant capacity of the genotype can be linked to its increased phenolic content.^32^, ^33^ Consistent with previous studies, DPPH values showed a positive correlation with total phenolic and flavonoid contents across the bean varieties.^9^ Dry pigmented beans exhibited higher FRAP antioxidant capacity compared to white beans, suggesting that their predominant antioxidant activity is mediated through reduction‐based mechanisms.

### Carotenoids, anthocyanins, proanthocyanins (PAs) and NEPAs

The total contents of carotenoids, anthocyanins, PAs and NEPAs from both raw and roasted bean flour are presented in Table 3. The total carotenoid content varied from 0.002 to 0.012 g kg^−1^ DW, with the highest concentration found to be ChickP (0.012 g kg^−1^ DW), which was stable (0.011 g kg^−1^ DW) after roasting. In contrast, the lowest concentration was found in WK‐2 (0.002 g kg^−1^ DW), with a 5% increase after roasting. There was no significant change among MAN, DRK and LRK after roasting, while there was an increase in the carotenoid content of MAY, CRAN‐1 and CRAN‐2 by 28%, 20% and 33% after roasting, respectively (*P* < 0.05). Carotenoids are well known to exert antioxidant and anti‐inflammatory properties, offering various health benefits, and inadequate intake may elevate the risk of depression.^34^

**Table 3:** Anthocyanin, proanthocyanin, non‐extractable polymeric proanthocyanin and total saponin of raw and roasted beans

	Anthocyanin (g kg^−1^ DW cyanidin‐3‐glucoside eq.)		Proanthocyanin (g kg^−1^ procyanidin B2 eq. DW)		NEPA (g kg^−1^ DW)		Saponin (g kg^−1^ oleanolic acid eq.)	
	Raw	Roasted	Raw	Roasted	Raw	Roasted	Raw	Roasted
OTE	n.d.	n.d.	0.537 ± 0.01	0.507 ± 0.02	0.031 ± 0.004	0.024 ± 0.003	1.65 ± 0.06	1.50 ± 0.04
GN	n.d.	n.d.	0.540 ± 0.03	0.517 ± 0.01	0.030 ± 0.003	0.027 ± 0.001	1.93 ± 0.08	1.60 ± 0.01
NAVY	n.d.	n.d.	0.520 ± 0.02	0.516 ± 0.02	0.046 ± 0.004	0.027 ± 0.004	1.98 ± 0.04	1.77 ± 0.04
WK‐1	n.d.	n.d.	0.652 ± 0.04	0.638 ± 0.06	0.023 ± 0.001	0.026 ± 0.005	0.63 ± 0.03	0.40 ± 0.02
WK‐2	n.d.	n.d.	0.757 ± 0.04	0.742 ± 0.05	0.047 ± 0.002	0.030 ± 0.002	0.50 ± 0.00	0.48 ± 0.00
MAN	n.d.	n.d.	0.595 ± 0.03	0.592 ± 0.02	0.024 ± 0.001	0.026 ± 0.001	0.76 ± 0.03	0.62 ± 0.04
MAY	n.d.	n.d.	0.572 ± 0.02	0.575 ± 0.04	0.032 ± 0.004	0.025 ± 0.001	0.49 ± 0.06	0.50 ± 0.03
CRAN‐1	0.003 ± 0.001	3.89 ± 1.74	0.743 ± 0.05	0.737 ± 0.03	0.122 ± 0.010	0.146 ± 0.015	0.47 ± 0.00	0.11 ± 0.03
CRAN‐2	0.057 ± 0.004	35.66 ± 7.97	1.458 ± 0.14	1.006 ± 0.06	0.137 ± 0.002	0.112 ± 0.008	1.81 ± 0.02	1.23 ± 0.02
LRK	0.035 ± 0.004	41.23 ± 7.57	0.998 ± 0.09	1.069 ± 0.02	0.133 ± 0.001	0.128 ± 0.003	0.97 ± 0.06	0.93 ± 0.01
DRK	0.039 ± 0.007	29.82 ± 2.50	0.884 ± 0.04	0.873 ± 0.05	0.113 ± 0.002	0.103 ± 0.004	1.09 ± 0.01	1.05 ± 0.01
ChickP	n.d.	n.d.	0.746 ± 0.04	0.767 ± 0.03	0.038 ± 0.002	0.037 ± 0.002	1.05 ± 0.04	0.67 ± 0.01
*P* value (raw *versus* roasted)	NS		NS		NS		**	

Data represent mean ± standard deviation (*n* = 3). All data are reported on a dry basis (DB). Data were analyzed by one‐way ANOVA according to an analysis of variance and Tukey's multiple mean comparison test (*P* < 0.05). ‘n.d.’ = not determined. Otebo: Samurai (OTE); Great Northern: Powderhorn (GN); Navy Alpena (NAVY); Mayacoba: Y1802‐9‐1 (MAY); White Kidney: WK1601‐1 (WK‐1); Cranberry: CR1801‐2‐2 (CRAN‐1); Manteca: Y1608‐07 (MAN); Chickpea: Sierra (ChickP); Red Hawk (DRK); Bellagio (CRAN‐2); Clouseau (LRK); ND whitetail (WK‐2). All analyses are measured in DW (dry weight). The significance level was checked between two pairs of raw and roasted at *P ≤ 0.05; **P ≤ 0.01; ***P ≤ 0.001; and NS = non‐significant.

**Table 4:** Correlations between total phenolics, FRAP, NEPAs and PAs in raw and roasted bean flours

Correlations	*R* ^2^	
	Raw	Roasted
Total phenolics *versus* FRAP	0.913	0.697
Total phenolics *versus* NEPAs	0.842	0.912
Total phenolics *versus* PAs	0.653	0.513
FRAP *versus* PAs	0.857	0.751
FRAP *versus* NEPAs	0.881	0.823
NEPAs *versus* PAs	0.799	0.782

All correlations were at *P* < 0.01.

**Table 5:** Nutritional indexes of bean cultivars

		OTE	GN	NAVY	WK‐1	WK‐2	MAN	May	CRAN‐1	CRAN‐2	LRK	DRK	ChickP	*P*
IA	Raw	1.25	2.08	1.15	1.28	1.12	0.91	1.03	1.19	1.21	1.31	1.17	0.29	**
	Roasted	1.25	2.12	1.15	1.35	1.21	0.91	1.12	1.21	1.23	1.39	1.18	0.30	
IT	Raw	2.55	4.34	2.61	2.88	2.65	1.93	2.18	2.58	2.68	2.85	2.50	0.59	*
	Roasted	2.59	4.41	2.60	2.98	2.75	1.91	2.37	2.53	2.74	3.01	2.54	0.59	
HH	Raw	0.78	0.43	0.82	0.70	0.81	1.00	0.89	0.76	0.77	0.71	0.80	3.43	*
	Roasted	0.78	0.43	0.82	0.66	0.74	1.00	0.83	0.75	0.76	0.67	0.80	3.40	
HPI	Raw	0.80	0.48	0.87	0.78	0.89	1.10	0.97	0.84	0.82	0.76	0.86	3.40	**
	Roasted	0.80	0.47	0.87	0.74	0.83	1.10	0.90	0.83	0.82	0.72	0.85	3.36	
EPA + DHA	Raw	0.15	0.16	0.13	0.12	0.06	0.41	0.14	0.15	0.08	0.06	0.12	0.05	NS
	Roasted	0.16	0.14	0.08	0.14	0.08	0.32	0.12	0.26	0.08	0.05	0.06	0.03	

IA, index of atherogenicity; IT, index of thrombogenicity; HH, hypocholesterolemic/hypercholesterolemic ratio; HPI, health‐promoting index; EPA + DHA, sum of eicosapentaenoic acid and docosahexaenoic acid.

*P ≤ 0.05; **P ≤ 0.01; ***P ≤ 0.001; NS, not significant (*P* > 0.05).

Anthocyanins are mostly present in dark beans as they contribute to the black/red color in those varieties.^30^, ^35^ The anthocyanin content in raw pigmented bean ranges from 0.003 to 0.057 g kg^−1^ DW, with the highest anthocyanin content found in CRAN‐2 with 0.057 ± 0.004 g kg^−1^ DW before roasting and 0.036 ± 0.008 g kg^−1^ DW after roasting, followed by DRK (0.039 ± 0.007 g kg^−1^ DW) before roasting and 0.030 ± 0.013 g kg^−1^ DW after roasting. We observed no significant differences in the anthocyanin content after roasting, confirming that dry heat roasting is a protective technology for this group of compounds. Xu and Chang^36^ reported a significant reduction in anthocyanins in black beans following wet thermal treatments (boiling and steaming), highlighting that the impact of heat on anthocyanin stability may depend on both the type of bean and the specific thermal processing conditions. In contrast, no significant differences in anthocyanin content were observed after roasting in our study, suggesting that dry heat roasting may act as a protective method for preserving these compounds. As expected, the concentration of anthocyanins in white/yellow beans and ChickP was very low or undetectable. It has been reported that Kabuli chickpeas are not typically a significant source of anthocyanins compared to pigmented beans and black beans, especially due to their white or beige‐colored seeds.^37^ Generally, anthocyanins are beneficial for their antidiabetic protective effects and protection against various non‐communicable diseases.^38^

The occurrence of PAs in dry beans is relatively sparse, as they are primarily found in pigmented grains and fruits.^8^ Among the different bean varieties, non‐pigmented beans had PA levels ranging from 0.540 to 0.757 g kg^−1^ DW before roasting and 0.507 to 0.742 g kg^−1^ DW after roasting. Pigmented beans exhibit higher PA concentrations, with CRAN‐2 showing the highest levels at 1.458 ± 0.14 g kg^−1^ DW before roasting and 1.006 ± 0.06 g kg^−1^ DW after roasting. The result is similar to that of the study by Dzomba *et al*.,^39^ who reported the order of variation in both anthocyanin and PA as brown (0.59 ± 0.01) > black (0.43 ± 0.01) > white (0.28 ± 0.01) using a differential method with a UV spectrophotometer; and Akond *et al*.,^35^ who reported that non‐pigmented beans exhibit a low concentration of PAs. PAs are significant for their potent antioxidant and anti‐inflammatory properties, which help reduce the risk of chronic diseases.^40^, ^41^

The NEPA content in different bean varieties remains understudied. Usually, after the extraction of monomeric and oligomeric PAs, a major portion of the polymeric PAs remains intact and is not considered for analysis.^41^ In the present study, the NEPA concentrations in unpigmented, raw‐bred bean varieties ranged from 2.32 to 4.62 g kg^−1^ before roasting and from 2.37 to 3.04 g kg^−1^ after roasting, without any significant change. In contrast, the pigmented, raw bean CRAN‐2 (13.69 ± 0.21 mg g^−1^) exhibited significantly higher NEPA levels, comparable to those of other pigmented varieties such as DRK (11.31 ± 0.16 g kg^−1^), CRAN‐1 (12.17 ± 0.95 g kg^−1^) and LRK (13.26 ± 0.14 g kg^−1^). These findings reinforce previous observations that pigmentation plays a key role in the availability of PAs in dry beans. For instance, Freixas Coutin *et al*.^42^ reported that cranberry beans contained higher concentrations of extractable PAs compared to non‐pigmented varieties. The relatively stable NEPA concentration after roasting suggests that they are more resistant to thermal degradation, and a complete quantification of PAs requires the determination of NEPAs.^41^

### Total saponin content

Results show up to 77% reduction in the saponin content after roasting (*P* < 0.05) (Table 3). For example, in CRAN‐2, WK‐1, ChickP and CRAN‐1, which were reduced by 32%, 36.5%, 36.2% and 76.6%, respectively; however, there were no significant differences between raw and roasted MAY^43^ under the roasting condition of 110 °C for 70 min. Studies have shown that saponins are sensitive to high temperatures and easily degraded during roasting. Saponins have been characterized as one of the non‐volatile compounds that contribute to the bitterness and off‐flavors in beans and other pulse foods.^44^ Given that saponins are a primary contributor to bitterness and are sensitive to high roasting temperatures, cultivars that exhibit a significant reduction in saponin content post‐roasting would be preferable for flour production aimed at minimizing bitterness.^45^ Therefore, based on this study, the cranberry bean samples are well suited for use as flour in product development due to their reduced saponin levels, which can enhance taste profiles and increase broader consumer appeal.

### Effect of roasting on fatty acids composition of dry beans

The fatty acid composition of beans was analyzed, revealing significant findings (Tables S1–S4, Supporting Information). Overall, the predominant fatty acids present in most bean flours were palmitic acid (C16:0), oleic acid (C18:1 Cis), stearic acid (C18:0), behenic acid (C22:0) and lignoceric acid (C24:0). The nutritional indexes of raw and roasted beans with their fatty acid profiles are presented in Table 5. Saturated fatty acids were the most abundant across all cultivars (*P* ≤ 0.001). Overall, *ca* 46% are monounsaturated fatty acids and *ca* 64% of the fatty acids found in both raw and roasted bean flour in the present varieties are saturated fatty acids with <1% short‐chain saturated fatty acids, <1% polyunsaturated fatty acids, <4% monounsaturated fatty acids and *ca* 95% are long‐chain fatty acids, except in chickpea that has 72% monounsaturated fatty acids and *ca* 28% saturated fatty acids. Roasting at 110 °C for 70 min did not significantly alter the composition of fatty acids (*P* ≤ 0.05), which aligns with Zhang *et al*.,^46^ who reported that light roasted peanut oil (180 °C for 10 min) has no significant effect on the fatty acid content compared to dark roasting of peanut oil (180 °C for 40 min) where there was a 38.29% reduction (*P* < 0.05).

The different bean cultivars exhibited variation in the levels of health‐promoting fatty acids and indexes. Among the various nutritional indexes calculated, the index of atherogenicity, index of thrombogenicity, hypocholesterolemic/hypercholesterolemic ratio^47^ and health‐promoting index were noteworthy in comparing the raw and roasted dry beans. The ratio of polyunsaturated fatty acids to saturated fatty acids was low across all samples, indicating that they had a higher concentration of saturated fatty acids. Roasted beans generally exhibited a slight increase in monounsaturated fatty acid content, while the levels of polyunsaturated fatty acids and the associated indexes remained relatively stable post‐roasting. These findings reinforce that dry heat helps retain fatty acids, thereby enhancing their contribution to nutritional quality.^48^ As a result, bean flours may play a supportive role in disease prevention and management.

### Total phytosterol content

In this study, stigmasterol, *β*‐sitosterol and fucosterol were identified by comparing their retention time and mass fragmentation pattern with pure standards (Table S5, supporting information). In addition, squalene was also separated within the 25 min run. Stigmasterol (0.15–0.11 to 0.54–1.04 g kg^−1^) and B‐sitosterol (0.42–1.62 to 0.66–2.36 g kg^−1^) in both raw and roasted samples, respectively, contributed the most to the total phytosterol levels in raw (24–30% and 46–67%) and roasted (21–33% and 46–69%) samples. This result agrees with the phytosterol content in other leguminous plants such as soy.^49^

**Figure 2:**
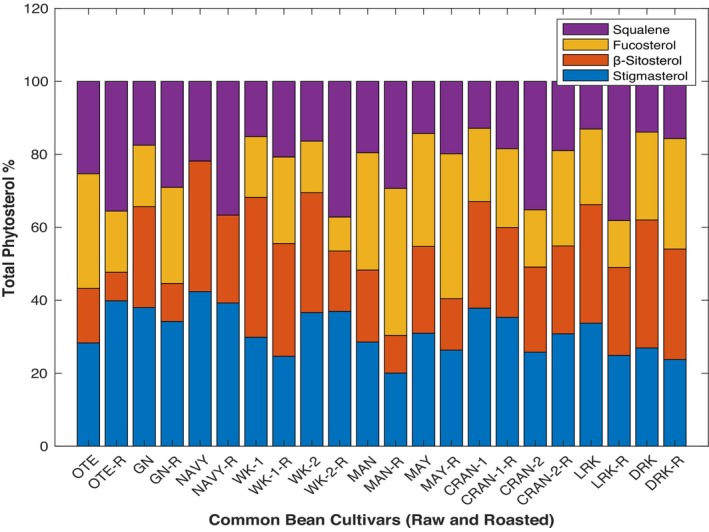
Phytosterol and squalene content of oil fractions extracted from 11 bean flours.

Roasting enhances the bioavailability of beneficial phytosterols, which are known to support cardiovascular health by lowering cholesterol levels.^50^ The phytosterol content significantly varies with roasting at 110 °C for 70 min. There was an increase in the availability of stigmasterol and *β*‐sitosterol (Fig. 2). NAVY, MAN and LRK stand out for their relatively high levels of squalene after roasting, with concentrations ranging from 0.10 to 0.43 and from 8.7 to 48.24 g kg^−1^, respectively. Squalene is known to have antioxidant and skin‐protective properties.^51^ Both NAVY and CRAN‐2 show an increase in the concentration of stigmasterol, but only CRAN‐2 had an increased concentration of *β*‐sitosterol after roasting. In contrast, MAY shows a decrease in concentration across all phytosterols identified after heating. The extent of the changes after roasting differs significantly, likely due to the time and temperatures used. Studies have shown that phytosterol content decreases in the first 0–20 min at 150 °C and increases when close to 60 min of roasting in pumpkin.^52^, ^53^ This suggests that thermal effects on phytosterols depend on oxidation and esterification reactions occurring in the bean oil during roasting.^54^ Additionally, the variation in phytosterol concentration might also be influenced by moisture loss during roasting, which can alter the extraction efficiency and solubility of the sample.^55^ Overall, these findings highlight the potential of roasted beans as a functional food source rich in heart‐healthy fats and bioactive compounds.

**Figure 3:**
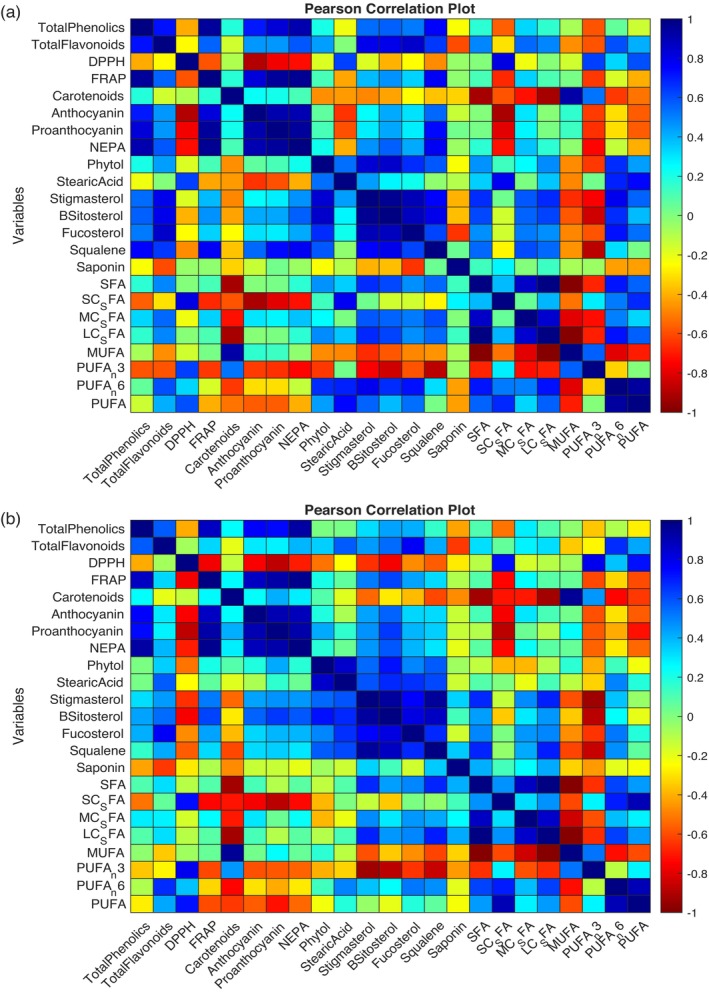
(a) Pearson correlation values of raw bean flours (at *P* < 0.05): total phenolic, total flavonoid, DPPH (2,2‐diphenyl‐1‐picrylhydrazyl); FRAP (ferric reducing antioxidant power); carotenoid; NEPA (non‐extractable polymeric proanthocyanins); anthocyanin; proanthocyanin; phytol; stearic acid; B‐sitosterol; stigmasterol; fucosterol; squalene; saponin; SFA (saturated fatty acids); SC_SFA (short‐chain saturated fatty acids); MC_SFA (medium‐chain saturated fatty acids); LC_SFA (long‐chain saturated fatty acids); MUFA (monounsaturated fatty acids); PUFA_n3 (omega‐3 polyunsaturated fatty acids); PUFA_n6 (omega‐6 polyunsaturated fatty acids); PUFA (polyunsaturated fatty acids). (b) Pearson correlation values between roasted bean flours (at *P* < 0.05): total phenolic, total flavonoid, DPPH (2,2‐diphenyl‐1‐picrylhydrazyl); FRAP (ferric reducing antioxidant power); carotenoid; NEPA (non‐extractable polymeric proanthocyanins); anthocyanin; proanthocyanin; phytol; stearic acid; B‐sitosterol; stigmasterol; fucosterol; squalene; saponin; SFA (saturated fatty acids); SC_SFA (short‐chain saturated fatty acids); MC_SFA (medium‐chain saturated fatty acids); LC_SFA (long‐chain saturated fatty acids); MUFA (monounsaturated fatty acids); PUFA_n3 (omega‐3 polyunsaturated fatty acids); PUFA_n6 (omega‐6 polyunsaturated fatty acids); PUFA (polyunsaturated fatty acids).

**Figure 4:**
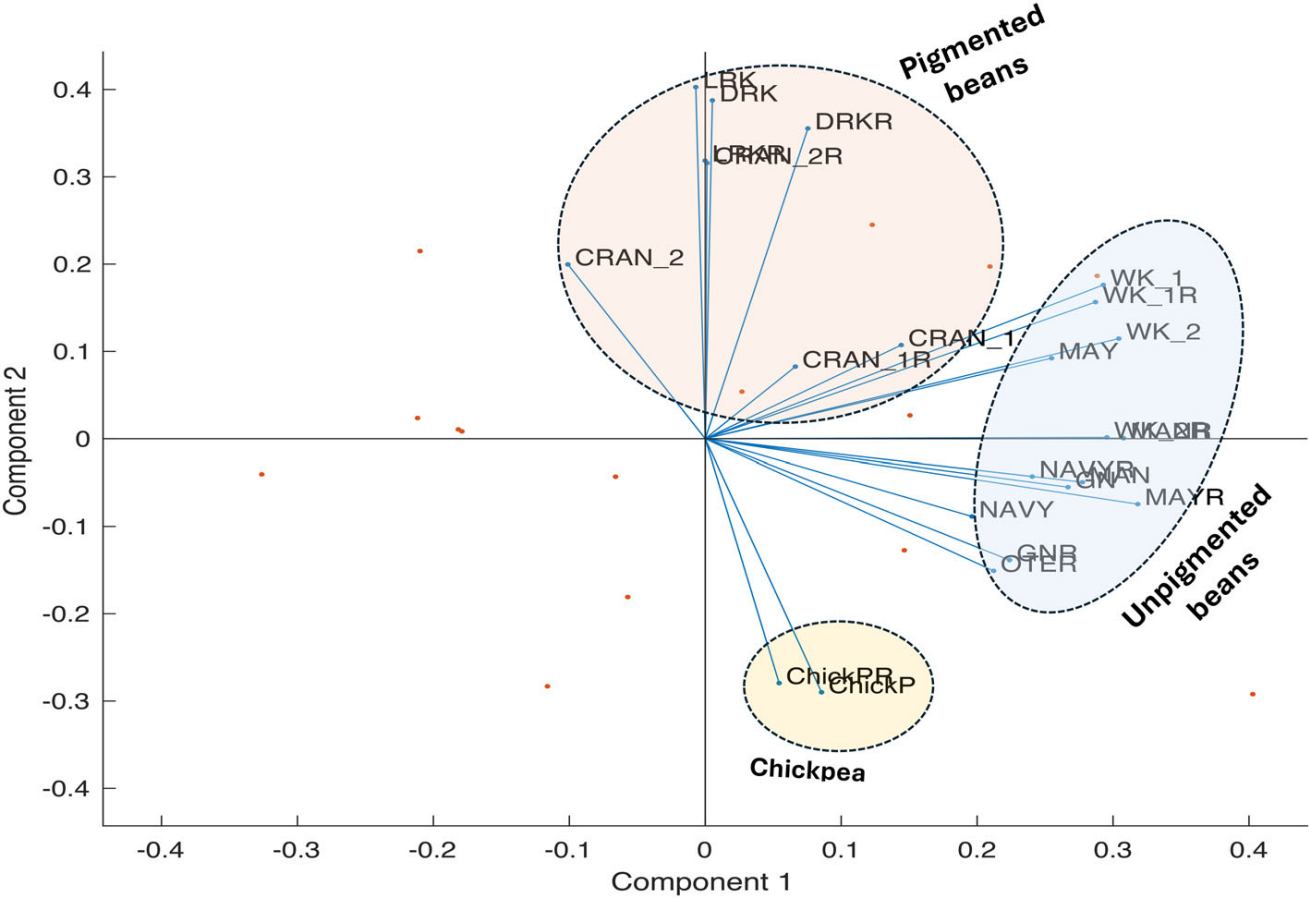
PCA showing pigmented beans, non‐pigmented beans and chickpea cultivars.

Pearson correlations in Fig. 3(a),(b) illustrate the association among phytochemicals, antioxidants, fatty acids, phytosterols and saponins, highlighting the interconnectedness of these bioactive compounds in dry beans. There was a strong correlation between total phenolics and FRAP, total phenolics, and NEPA (*R* ^2^ = 0.82 and 0.87, at 0.01 significant levels, respectively). FRAP also had a strong correlation with anthocyanins, PAs and NEPA (*R* ^2^ = 0.50 at 0.05, and *R* ^2^ = 0.81 and *R* ^2^ = 0.85 at 0.01 significant levels, respectively). Likewise, total flavonoids and total phenolics had a positive correlation with phytosterols. Raw and roasted samples had similar correlation values (Fig. 3(a),(b)). PCA elucidated distinct clusters among bean varieties based on their phytochemical and antioxidant profiles, as seen in Fig. 4. The factor scores of the bean cultivars were used to form three clusters distributed based on their individual phytochemicals and antioxidant concentrations. The bean cultivars clustered into pigmented beans, unpigmented beans and chickpeas, which correlates with their overall phytochemical and antioxidant activities. PCA helps to understand previous studies that reported that pigmented bean cultivars with blue, red, black and pink colors have high phytochemical contents and antioxidant activities.^56^

## CONCLUSION

This study provides a comprehensive evaluation of phytochemicals, fatty acids, saponins and antioxidant concentrations of 11 dry bean varieties and one chickpea variety, offering new insights into their bioactive and nutritional attributes and potential targets for functional food applications.

The findings confirm that common beans are rich in bioactive compounds, including phenolics, flavonoids, NEPAs and phytosterols, all of which contribute to their high antioxidant capacity. Pigmented varieties exhibit higher concentrations of these compounds compared to non‐pigmented ones, reinforcing their value as health‐promoting ingredients.

Importantly, the study highlights the role of roasting as a thermal processing technology to preserve and enhance, in particular cases, key nutritional attributes when applied under mild‐controlled conditions (110 °C for 70 min). While roasting resulted in a moderate reduction of thermally sensitive compounds such as anthocyanins, it improved the bioavailability of phytosterols in the majority of cultivars studied. In the same way, roasting maintained the fatty acid composition and health indexes. Utilizing pigmented dry bean cultivars for flour production and optimizing roasting parameters based on specific cultivar characteristics may lead to the development of bean‐based flours with improved health‐promoting and nutritional quality. The data support the use of bean flours as key ingredients in the formulation of functional foods aimed at promoting health benefits while reducing the persistence of chronic disorders. This work offers a valuable framework for maximizing the health‐promoting properties of dry beans in both culinary and industrial contexts.

## Supporting information


**Data S1:** Supporting Information.

## Data Availability

The data that supports the findings of this study are available in the supplementary material of this article.
