# Supplementary material for: Enhancing phytochemical composition and nutritional profiles in dry bean varieties through roasting
Source: J Sci Food Agric. 2025 Aug 12;105(15):8694–705. doi: 10.1002/jsfa.70106 (PMC12595396; doi:10.1002/jsfa.70106)
Supplement: Supplementary file 1 — Data S1: Supporting Information. [file JSFA-105-8694-s001.docx]

**Table S1.** Saturated fatty acid composition in Raw Samples of 12 Bean Varieties (% of total fatty acids)

|  | **OTE** | **GN** | **NAVY** | **WK-1** | **WK-2** | **MAN** | **MAY** | **CRAN-1** | **CRAN-2** | **LRK** | **DRK** | **ChickP** | **P-value** |
| --- | --- | --- | --- | --- | --- | --- | --- | --- | --- | --- | --- | --- | --- |
| **C4:0** | 0.49 ± 0.08 | 0.47 ± 0.00 | 0.57 ± 0.12 | 0.37 ± 0.04 | 0.18 ± 0.00 | 0.35 ± 0.03 | 0.47 ± 0.03 | 0.41 ± 0.07 | 0.28 ± 0.19 | 0.18 ± 0.06 | 0.11 ± 0.03 | 0.16 ± 0.01 | *** |
| **C6:0** | 0.52 ± 0.03 | 0.27 ± 0.03 | 0.34 ± 0.04 | 0.43 ± 0.01 | 1.29 ± 0.02 | 0.38 ± 0.07 | 0.41 ± 0.05 | 0.47 ± 0.13 | 0.81 ± 0.19 | 0.66 ± 0.16 | 0.65 ± 0.08 | 0.18 ± 0.01 | *** |
| **C8:0** | 0.37 ± 0.02 | 0.15 ± 0.01 | 0.05 ± 0.00 | 0.20 ± 0.01 | 0.47 ± 0.02 | 0.26 ± 0.07 | 0.26 ± 0.01 | 0.20 ± 0.02 | 0.31 ± 0.08 | 0.25 ± 0.04 | 0.19 ± 0.02 | 0.06 ± 0.01 | *** |
| **C10:0** | 0.53 ± 0.05 | 0.27 ± 0.00 | 0.19 ± 0.01 | 0.35 ± 0.01 | 0.52 ± 0.02 | 0.30 ± 0.04 | 0.36 ± 0.04 | 0.43 ± 0.08 | 0.45 ± 0.02 | 0.47 ± 0.02 | 0.36 ± 0.03 | 0.03 ± 0.01 | *** |
| **C11:0** | 0.83 ± 0.14 | 0.64 ± 0.10 | 0.45 ± 0.07 | 0.59 ± 0.02 | 0.65 ± 0.09 | 0.50 ± 0.05 | 0.68 ± 0.01 | 0.67 ± 0.12 | 0.48 ± 0.20 | 0.36 ± 0.01 | 0.37 ± 0.09 | 0.02 ± 0.01 | *** |
| **C12:0** | 0.88 ± 0.08 | 0.38 ± 0.07 | 0.23 ± 0.00 | 0.33 ± 0.04 | 0.21 ± 0.00 | 0.31 ± 0.09 | 0.47 ± 0.02 | 0.44 ± 0.12 | 0.38 ± 0.15 | 0.30 ± 0.07 | 0.41 ± 0.17 | 0.09 ± 0.01 | *** |
| **C14:0** | 1.42 ± 0.02 | 1.09 ± 0.16 | 0.76 ± 0.07 | 0.91 ± 0.05 | 0.59 ± 0.03 | 0.69 ± 0.10 | 1.22 ± 0.23 | 1.16 ± 0.24 | 0.82 ± 0.07 | 0.68 ± 0.00 | 0.58 ± 0.06 | 0.25 ± 0.00 | *** |
| **C15:0** | 0.99 ± 0.10 | 1.06 ± 0.08 | 0.43 ± 0.13 | 0.43 ± 0.03 | 0.39 ± 0.02 | 0.36 ± 0.02 | 0.48 ± 0.04 | 0.59 ± 0.04 | 0.42 ± 0.04 | 0.40 ± 0.05 | 0.36 ± 0.00 | 0.12 ± 0.01 | *** |
| **C16:0** | 40.77 ± 0.27 | 51.35 ± 0.49 | 40.95 ± 1.20 | 42.89 ± 0.82 | 39.15 ± 0.08 | 37.20 ± 0.28 | 37.56 ± 0.95 | 39.66 ± 1.05 | 41.97 ± 0.53 | 45.78 ± 0.04 | 43.92 ± 0.19 | 20.53 ± 0.34 | *** |
| **C17:0** | 1.11 ± 0.09 | 0.98 ± 0.05 | 0.71 ± 0.02 | 0.94 ± 0.08 | 0.97 ± 0.07 | 1.01 ± 0.24 | 0.87 ± 0.05 | 1.17 ± 0.05 | 0.83 ± 0.02 | 0.74 ± 0.01 | 0.73 ± 0.02 | 0.15 ± 0.00 | *** |
| **C18:0** | 8.41 ± 0.30 | 10.23 ± 0.70 | 8.87 ± 0.00 | 7.62 ± 0.36 | 10.04 ± 0.24 | 6.86 ± 0.35 | 6.93 ± 0.01 | 8.05 ± 0.12 | 7.74 ± 0.11 | 6.16 ± 0.13 | 5.65 ± 0.03 | 3.25 ± 0.07 | *** |
| **C20:0** | 2.00 ± 0.03 | 2.12 ± 0.01 | 2.31 ± 0.03 | 2.27 ± 0.01 | 2.65 ± 0.00 | 1.93 ± 0.05 | 2.03 ± 0.00 | 2.26 ± 0.02 | 1.95 ± 0.05 | 1.81 ± 0.08 | 1.74 ± 0.01 | 1.66 ± 0.01 | NS |
| **C21:0** | 0.78 ± 0.02 | 0.77 ± 0.02 | 0.94 ± 0.02 | 1.01 ± 0.12 | 0.16 ± 0.01 | 0.92 ± 0.00 | 1.07 ± 0.00 | 0.97 ± 0.05 | 0.73 ± 0.03 | 0.75 ± 0.01 | 0.75 ± 0.02 | 0.02 ± 0.00 | *** |
| **C22:0** | 2.81 ± 0.06 | 2.96 ± 0.22 | 3.94 ± 0.68 | 4.79 ± 0.24 | 4.84 ± 0.30 | 4.41 ± 0.38 | 5.66 ± 0.42 | 5.12 ± 1.05 | 3.75 ± 0.05 | 3.12 ± 0.07 | 3.13 ± 0.15 | 0.74 ± 0.02 | *** |
| **C23:0** | 0.07 ± 0.01 | 0.13 ± 0.02 | 0.12 ± 0.01 | 0.19 ± 0.02 | 0.09 ± 0.01 | 0.19 ± 0.02 | 0.23 ± 0.01 | 0.15 ± 0.00 | 0.11 ± 0.01 | 0.12 ± 0.01 | 0.11 ± 0.00 | 0.01 ± 0.00 | *** |
| **C24:0** | 1.63 ± 0.03 | 2.30 ± 0.03 | 2.73 ± 0.03 | 2.62 ± 0.09 | 2.44 ± 0.21 | 2.54 ± 0.31 | 2.56 ± 0.08 | 3.16 ± 0.05 | 2.90 ± 0.13 | 2.68 ± 0.06 | 2.42 ± 0.24 | 0.32 ± 0.01 | *** |
| **Ʃ SFA** | 63.62 | 75.17 | 63.6 | 65.95 | 64.64 | 61.27 | 58.22 | 64.91 | 63.92 | 64.46 | 61.49 | 27.6 | *** |
| **Ʃ SC-SFA** | 0.49 | 0.47 | 0.57 | 0.37 | 0.18 | 0.47 | 0.35 | 0.41 | 0.28 | 0.18 | 0.11 | 0.16 | *** |
| **Ʃ MC-SFA** | 3.14 | 1.71 | 1.27 | 1.91 | 3.15 | 2.19 | 1.76 | 2.2 | 2.43 | 2.04 | 1.99 | 0.39 | *** |
| **Ʃ LC-SFA** | 59.99 | 72.99 | 61.76 | 63.67 | 61.31 | 58.61 | 56.11 | 62.3 | 61.22 | 62.24 | 59.39 | 27.05 | *** |

*Data represent means ± standard deviation (n = 2). (Otebo: Samurai (OTE); Great Northern: Powderhorn) (GN); Navy Alpena (NA); Mayacoba: Y1802-9-1 (MAY); White Kidney:WK1601-1 (WK); Cranberry: CR1801-2-2 (CRB); Manteca: Y1608-07 (MAN); Chickpea: Sierra (ChickP); Red Hawk (RH); Bellagio (BELL); Clouseau (CLOU);ND whitetail (WT)); ΣSFA: Sum of saturated fatty acid, ΣSC-FA: sum of short chain saturated fatty acid, ΣMC-FA: sum of medium chain saturated fatty acid, ΣLC-FA long chain saturated fatty acid. The significance level was checked between the varieties at P ≤0.05,*; P ≤ 0.01, **; P ≤ 0.001,***; and NS=nonsignificant.*

**Table S2.** Saturated fatty acid composition in Roasted Samples of 12 Bean Varieties (% of total fatty acids)

|  | **OTE-R** | **GN-R** | **NAVY-R** | **WK-1-R** | **WK-2-R** | **MAN-R** | **MAY-R** | **CRAN-1-R** | **CRAN-2-R** | **LRK-R** | **DRK-R** | **ChickP-R** | **P-value** |
| --- | --- | --- | --- | --- | --- | --- | --- | --- | --- | --- | --- | --- | --- |
| **C4:0** | 0.47 ± 0.09 | 0.50 ± 0.06 | 0.31 ± 0.01 | 0.47 ± 0.11 | 0.25 ± 0.05 | 0.51 ± 0.19 | 0.43 ± 0.06 | 0.34 ± 0.05 | 0.27 ± 0.02 | 0.22 ± 0.10 | 0.19 ± 0.11 | 0.17 ± 0.00 | *** |
| **C6:0** | 0.60 ± 0.08 | 0.49 ± 0.08 | 0.26 ± 0.01 | 0.36 ± 0.08 | 0.78 ± 0.51 | 0.27 ± 0.03 | 0.78 ± 0.10 | 0.50 ± 0.10 | 0.81 ± 0.14 | 0.94 ± 0.08 | 0.92 ± 0.00 | 0.19 ± 0.05 | *** |
| **C8:0** | 0.35 ± 0.02 | 0.05 ± 0.01 | 0.03 ± 0.00 | 0.15 ± 0.00 | 0.35 ± 0.21 | 0.18 ± 0.03 | 0.28 ± 0.02 | 0.21 ± 0.00 | 0.33 ± 0.08 | 0.37 ± 0.05 | 0.36 ± 0.05 | 0.06 ± 0.02 | *** |
| **C10:0** | 0.48 ± 0.03 | 0.28 ± 0.04 | 0.27 ± 0.02 | 0.28 ± 0.05 | 0.54 ± 0.01 | 0.35 ± 0.01 | 0.53 ± 0.05 | 0.38 ± 0.06 | 0.42 ± 0.02 | 0.29 ± 0.06 | 0.36 ± 0.04 | 0.03 ± 0.01 | *** |
| **C11:0** | 0.72 ± 0.10 | 0.75 ± 0.11 | 0.40 ± 0.04 | 0.53 ± 0.13 | 0.47 ± 0.13 | 0.55 ± 0.00 | 0.82 ± 0.08 | 0.61 ± 0.15 | 0.54 ± 0.03 | 0.30 ± 0.03 | 0.44 ± 0.05 | 0.02 ± 0.01 | *** |
| **C12:0** | 0.86 ± 0.25 | 0.42 ± 0.05 | 0.20 ± 0.04 | 0.31 ± 0.01 | 0.38 ± 0.23 | 0.47 ± 0.13 | 0.49 ± 0.09 | 0.40 ± 0.04 | 0.26 ± 0.02 | 0.21 ± 0.01 | 0.26 ± 0.01 | 0.10 ± 0.00 | *** |
| **C14:0** | 1.29 ± 0.32 | 1.18 ± 0.08 | 0.71 ± 0.01 | 0.95 ± 0.00 | 0.76 ± 0.01 | 0.98 ± 0.18 | 1.14 ± 0.15 | 1.13 ± 0.10 | 0.66 ± 0.06 | 0.63 ± 0.05 | 0.67 ± 0.07 | 0.26 ± 0.01 | *** |
| **C15:0** | 1.08 ± 0.23 | 1.14 ± 0.09 | 0.39 ± 0.06 | 0.40 ± 0.09 | 0.48 ± 0.01 | 0.48 ± 0.09 | 0.50 ± 0.01 | 0.47 ± 0.12 | 0.36 ± 0.01 | 0.37 ± 0.03 | 0.35 ± 0.02 | 0.12 ± 0.01 | *** |
| **C16:0** | 41.55 ± 0.70 | 51.41 ± 0.49 | 41.69 ± 0.21 | 44.19 ± 0.02 | 41.24 ± 0.76 | 36.45 ± 0.09 | 39.03 ± 0.63 | 40.74 ± 0.27 | 42.67 ± 0.20 | 47.47 ± 0.24 | 43.62 ± 0.13 | 20.69 ± 0.23 | *** |
| **C17:0** | 1.06 ± 0.09 | 1.06 ± 0.01 | 0.70 ± 0.07 | 0.93 ± 0.08 | 0.88 ± 0.03 | 0.92 ± 0.03 | 0.99 ± 0.15 | 1.05 ± 0.09 | 0.78 ± 0.02 | 0.74 ± 0.06 | 0.70 ± 0.01 | 0.15 ± 0.01 | *** |
| **C18:0** | 7.73 ± 0.00 | 9.65 ± 0.10 | 8.70 ± 0.34 | 7.09 ± 0.15 | 9.78 ± 0.17 | 6.77 ± 0.50 | 6.91 ± 0.09 | 7.56 ± 0.17 | 7.51 ± 0.01 | 5.73 ± 0.21 | 5.64 ± 0.03 | 3.22 ± 0.00 | *** |
| **C20:0** | 1.81 ± 0.16 | 2.08 ± 0.00 | 2.21 ± 0.04 | 2.37 ± 0.03 | 1.25 ± 1.77 | 1.88 ± 0.05 | 2.04 ± 0.01 | 2.21 ± 0.04 | 1.97 ± 0.02 | 1.58 ± 0.02 | 1.79 ± 0.00 | 1.64 ± 0.07 | *** |
| **C21:0** | 0.78 ± 0.01 | 0.80 ± 0.04 | 0.86 ± 0.04 | 0.95 ± 0.04 | 0.43 ± 0.42 | 0.94 ± 0.01 | 1.07 ± 0.07 | 0.98 ± 0.05 | 0.74 ± 0.04 | 0.72 ± 0.02 | 0.77 ± 0.01 | 0.02 ± 0.00 | *** |
| **C22:0** | 2.80 ± 0.03 | 2.70 ± 0.01 | 3.86 ± 0.00 | 4.86 ± 0.64 | 4.81 ± 0.53 | 5.01 ± 0.21 | 4.67 ± 0.17 | 4.80 ± 0.86 | 3.94 ± 0.05 | 3.04 ± 0.18 | 3.22 ± 0.01 | 0.76 ± 0.01 | *** |
| **C23:0** | 0.08 ± 0.00 | 0.10 ± 0.01 | 0.15 ± 0.02 | 0.19 ± 0.07 | 0.17 ± 0.12 | 0.22 ± 0.03 | 0.18 ± 0.00 | 0.17 ± 0.03 | 0.09 ± 0.01 | 0.10 ± 0.01 | 0.10 ± 0.00 | 0.01 ± 0.00 | *** |
| **C24:0** | 1.63 ± 0.01 | 2.42 ± 0.04 | 2.27 ± 0.40 | 2.69 ± 0.04 | 2.67 ± 0.10 | 2.41 ± 0.19 | 2.59 ± 0.01 | 3.09 ± 0.04 | 2.95 ± 0.06 | 2.59 ± 0.27 | 2.50 ± 0.02 | 0.34 ± 0.01 | *** |
| **Ʃ SFA** | 63.3 | 75.05 | 63 | 66.73 | 65.22 | 62.46 | 58.4 | 64.61 | 64.3 | 65.31 | 61.89 | 27.77 | *** |
| **Ʃ SC-SFA** | 0.47 | 0.5 | 0.31 | 0.47 | 0.25 | 0.43 | 0.51 | 0.34 | 0.27 | 0.22 | 0.19 | 0.17 | ** |
| **Ʃ MC-SFA** | 3.02 | 1.99 | 1.16 | 1.63 | 2.51 | 2.9 | 1.82 | 2.09 | 2.35 | 2.11 | 2.34 | 0.4 | *** |
| **Ʃ LC-SFA** | 59.81 | 72.55 | 61.53 | 64.63 | 62.45 | 59.13 | 56.07 | 62.18 | 61.68 | 62.97 | 59.37 | 27.2 | *** |

*Data represent means ± standard deviation (n = 2). (Otebo: Samurai (OTE); Great Northern: Powderhorn) (GN); Navy Alpena (NA); Mayacoba: Y1802-9-1 (MAY); White Kidney:WK1601-1 (WK); Cranberry: CR1801-2-2 (CRB); Manteca: Y1608-07 (MAN); Chickpea: Sierra (ChickP); Red Hawk (RH); Bellagio (BELL); Clouseau (CLOU); ND whitetail (WT)); ΣSFA: Sum of saturated fatty acid, ΣSC-FA: sum of short chain saturated fatty acid, ΣMC-FA: sum of medium chain saturated fatty acid, ΣLC-FA long chain saturated fatty acid are reported in %. The significance level was checked between the varieties at P ≤0.05,*; P ≤ 0.01, **; P ≤ 0.001,***; and NS=nonsignificant.*

**Table S3.** Monounsaturated fatty acid composition in Raw and Roasted Samples of 12 Beans Varieties (% of total fatty acids)

| **Raw** | **OTE** | **GN** | **NAVY** | **WK-1** | **WK-2** | **MAN** | **MAY** | **CRAN-1** | **CRAN-2** | **LRK** | **DRK** | **ChickP** | **P-value** |
| --- | --- | --- | --- | --- | --- | --- | --- | --- | --- | --- | --- | --- | --- |
| **C14:1** | 0.27 ± 0.01 | 0.21 ± 0.04 | 0.18 ± 0.01 | 0.47 ± 0.07 | 0.14 ± 0.04 | 0.42 ± 0.09 | 0.39 ± 0.06 | 0.35 ± 0.08 | 0.21 ± 0.03 | 0.20 ± 0.05 | 0.18 ± 0.07 | 0.00 ± 0.00 | *** |
| **C16:1** | 1.14 ± 0.06 | 0.77 ± 0.09 | 0.86 ± 0.00 | 1.21 ± 0.17 | 1.39 ± 0.04 | 1.51 ± 0.07 | 1.51 ± 0.07 | 1.52 ± 0.16 | 1.22 ± 0.05 | 0.95 ± 0.00 | 0.95 ± 0.03 | 0.41 ± 0.01 | *** |
| **C17:1** | 0.44 ± 0.06 | 0.45 ± 0.14 | 0.25 ± 0.00 | 0.39 ± 0.09 | 0.52 ± 0.14 | 0.46 ± 0.03 | 0.51 ± 0.09 | 0.56 ± 0.01 | 0.73 ± 0.08 | 0.68 ± 0.07 | 0.62 ± 0.17 | 0.17 ± 0.00 | *** |
| **C18:1 Trans** | 0.23 ± 0.02 | 0.18 ± 0.04 | 0.18 ± 0.19 | 0.30 ± 0.19 | 0.28 ± 0.04 | 0.17 ± 0.04 | 0.29 ± 0.04 | 0.41 ± 0.11 | 0.19 ± 0.01 | 0.14 ± 0.02 | 0.13 ± 0.02 | 0.06 ± 0.01 | *** |
| **C18:1 Cis** | 31.66 ± 0.56 | 19.86 ± 0.23 | 31.98 ± 0.62 | 27.90 ± 0.05 | 30.14 ± 0.21 | 35.48 ± 1.08 | 31.44 ± 0.65 | 28.30 ± 0.28 | 31.30 ± 0.56 | 30.99 ± 0.03 | 34.11 ± 0.66 | 70.24 ± 0.45 | *** |
| **C20:1n9** | 0.43 ± 0.01 | 0.52 ± 0.01 | 0.51 ± 0.03 | 0.60 ± 0.00 | 0.59 ± 0.00 | 0.70 ± 0.02 | 0.74 ± 0.03 | 0.59 ± 0.04 | 0.46 ± 0.00 | 0.48 ± 0.01 | 0.50 ± 0.01 | 0.02 ± 0.00 | *** |
| **C22:1n9** | 0.04 ± 0.01 | 0.10 ± 0.03 | 0.11 ± 0.04 | 0.19 ± 0.02 | 0.02 ± 0.00 | 0.14 ± 0.02 | 0.19 ± 0.02 | 0.17 ± 0.06 | 0.03 ± 0.00 | 0.03 ± 0.03 | 0.03 ± 0.00 | 0.02 ± 0.00 | *** |
| **C24:1n9** | 0.11 ± 0.01 | 0.06 ± 0.00 | 0.05 ± 0.01 | 0.04 ± 0.01 | 0.02 ± 0.00 | 0.04 ± 0.01 | 0.06 ± 0.01 | 0.03 ± 0.00 | 0.03 ± 0.00 | 0.02 ± 0.00 | 0.03 ± 0.01 | 0.12 ± 0.01 | *** |
| **Ʃ MUFA** | 34.31 | 22.15 | 34.11 | 31.1 | 33.09 | 38.93 | 35.13 | 31.93 | 34.17 | 33.5 | 36.55 | 71.05 | *** |
|  |  |  |  |  |  |  |  |  |  |  |  |  |  |
| **Roasted** | **OTE-R** | **GN-R** | **NAVY-R** | **WK-1-R** | **WK-2-R** | **MAN-R** | **MAY-R** | **CRAN-1-R** | **CRAN-2-R** | **LRK-R** | **DRK-R** | **ChickP-R** |  |
| **C14:1** | 0.25 ± 0.03 | 0.25 ± 0.01 | 0.13 ± 0.09 | 0.49 ± 0.12 | 0.22 ± 0.02 | 0.52 ± 0.17 | 0.44 ± 0.09 | 0.31 ± 0.06 | 0.15 ± 0.04 | 0.17 ± 0.13 | 0.15 ± 0.02 | 0.00 ± 0.00 | *** |
| **C16:1** | 1.09 ± 0.10 | 0.80 ± 0.03 | 0.73 ± 0.08 | 1.21 ± 0.08 | 1.46 ± 0.04 | 1.42 ± 0.04 | 1.62 ± 0.10 | 1.50 ± 0.07 | 1.16 ± 0.00 | 0.89 ± 0.02 | 0.98 ± 0.03 | 0.42 ± 0.01 | *** |
| **C17:1** | 0.39 ± 0.06 | 0.54 ± 0.14 | 0.24 ± 0.01 | 0.43 ± 0.13 | 0.59 ± 0.11 | 0.51 ± 0.11 | 0.44 ± 0.10 | 0.56 ± 0.21 | 0.68 ± 0.09 | 0.39 ± 0.02 | 0.58 ± 0.04 | 0.18 ± 0.01 | *** |
| **C18:1 Trans** | 0.25 ± 0.07 | 0.13 ± 0.03 | 0.15 ± 0.01 | 0.17 ± 0.07 | 0.30 ± 0.01 | 0.20 ± 0.06 | 0.25 ± 0.06 | 0.48 ± 0.12 | 0.12 ± 0.02 | 0.11 ± 0.02 | 0.08 ± 0.01 | 0.05 ± 0.02 | *** |
| **C18:1 Cis** | 31.99 ± 1.02 | 19.99 ± 0.40 | 32.73 ± 1.01 | 27.10 ± 0.33 | 28.90 ± 0.29 | 34.25 ± 0.94 | 31.24 ± 0.78 | 28.74 ± 0.19 | 31.22 ± 0.15 | 30.70 ± 0.27 | 33.91 ± 0.27 | 70.13 ± 0.46 | *** |
| **C20:1n9** | 0.45 ± 0.02 | 0.54 ± 0.02 | 0.54 ± 0.01 | 0.65 ± 0.02 | 0.60 ± 0.03 | 0.70 ± 0.01 | 0.77 ± 0.00 | 0.62 ± 0.03 | 0.50 ± 0.01 | 0.49 ± 0.02 | 0.53 ± 0.00 | 0.03 ± 0.01 | *** |
| **C22:1n9** | 0.04 ± 0.00 | 0.06 ± 0.00 | 0.10 ± 0.01 | 0.12 ± 0.04 | 0.06 ± 0.06 | 0.16 ± 0.01 | 0.13 ± 0.00 | 0.15 ± 0.06 | 0.03 ± 0.00 | 0.04 ± 0.00 | 0.03 ± 0.00 | 0.02 ± 0.00 | *** |
| **C24:1n9** | 0.10 ± 0.01 | 0.06 ± 0.00 | 0.03 ± 0.01 | 0.04 ± 0.00 | 0.01 ± 0.01 | 0.04 ± 0.01 | 0.04 ± 0.00 | 0.03 ± 0.01 | 0.02 ± 0.00 | 0.02 ± 0.00 | 0.02 ± 0.00 | 0.07 ± 0.00 | *** |
| **Ʃ MUFA** | 34.55 | 22.36 | 34.65 | 30.21 | 32.15 | 37.79 | 34.93 | 32.39 | 33.87 | 32.82 | 36.28 | 70.89 | *** |

*Data represent means ± standard deviation (n = 2). (Otebo: Samurai (OTE); Great Northern: Powderhorn) (GN); Navy Alpena (NA); Mayacoba: Y1802-9-1 (MAY); White Kidney: WK1601-1 (WK); Cranberry: CR1801-2-2 (CRB); Manteca: Y1608-07 (MAN); Chickpea: Sierra (ChickP); Red Hawk (RH); Bellagio (BELL); Clouseau (CLOU); ND whitetail (WT)); ΣMUFA: sum of monounsaturated fatty acid, MUFA are reported in %. The significance level was checked between the varieties at P ≤0.05,*; P ≤ 0.01, **; P ≤ 0.001,***; and NS=nonsignificant.*

**Table S4.** Polyunsaturated fatty acid composition in Raw and Roasted Samples of 12 Beans Varieties (% of total fatty acids)

|  | **OTE** | **GN** | **NAVY** | **WK-1** | **WK-2** | **MAN** | **MAY** | **CRAN-1** | **CRAN-2** | **LRK** | **DRK** | **ChickP** | **P-value** |
| --- | --- | --- | --- | --- | --- | --- | --- | --- | --- | --- | --- | --- | --- |
| **C18:2 Trans** | 0.21 ± 0.00 | 0.11 ± 0.02 | 0.09 ± 0.03 | 0.10 ± 0.02 | 0.05 ± 0.05 | 0.04 ± 0.06 | 0.12 ± 0.03 | 0.13 ± 0.03 | 0.08 ± 0.01 | 0.07 ± 0.01 | 0.05 ± 0.00 | 0.00 ± 0.00 | *** |
| **C18:2 Cis** | 0.41 ± 0.07 | 0.44 ± 0.05 | 0.32 ± 0.10 | 0.36 ± 0.12 | 0.38 ± 0.02 | 0.31 ± 0.02 | 0.49 ± 0.19 | 0.56 ± 0.28 | 0.32 ± 0.05 | 0.34 ± 0.05 | 0.35 ± 0.03 | 0.21 ± 0.01 | *** |
| **C18:3n6** | 0.09 ± 0.01 | 0.10 ± 0.01 | 0.10 ± 0.01 | 0.15 ± 0.00 | 0.18 ± 0.00 | 0.18 ± 0.01 | 0.16 ± 0.01 | 0.13 ± 0.02 | 0.10 ± 0.00 | 0.11 ± 0.00 | 0.11 ± 0.00 | 0.00 ± 0.00 | *** |
| **C18:3n3** | 0.16 ± 0.01 | 0.18 ± 0.00 | 0.13 ± 0.01 | 0.14 ± 0.00 | 0.27 ± 0.02 | 0.14 ± 0.00 | 0.16 ± 0.01 | 0.12 ± 0.01 | 0.11 ± 0.01 | 0.13 ± 0.01 | 0.11 ± 0.01 | 0.77 ± 0.01 | *** |
| **C20:2n6** | 0.28 ± 0.05 | 0.42 ± 0.10 | 0.40 ± 0.18 | 0.62 ± 0.14 | 0.28 ± 0.04 | 0.43 ± 0.20 | 0.68 ± 0.30 | 0.54 ± 0.38 | 0.27 ± 0.02 | 0.31 ± 0.06 | 0.27 ± 0.01 | 0.16 ± 0.02 | *** |
| **C20:3n6** | 0.03 ± 0.00 | 0.25 ± 0.17 | 0.25 ± 0.32 | 0.55 ± 0.09 | 0.04 ± 0.01 | 0.34 ± 0.33 | 0.64 ± 0.28 | 0.38 ± 0.46 | 0.04 ± 0.02 | 0.05 ± 0.02 | 0.03 ± 0.01 | 0.02 ± 0.01 | *** |
| **C20:3n3** | 0.22 ± 0.00 | 0.36 ± 0.00 | 0.17 ± 0.00 | 0.09 ± 0.00 | 0.07 ± 0.00 | 0.23 ± 0.00 | 0.35 ± 0.00 | 0.30 ± 0.00 | 0.11 ± 0.00 | 0.09 ± 0.00 | 0.08 ± 0.00 | 0.01 ± 0.00 | *** |
| **C20:4n6** | 0.47 ± 0.00 | 0.63 ± 0.00 | 0.69 ± 0.00 | 0.78 ± 0.02 | 0.90 ± 0.06 | 0.73 ± 0.06 | 0.81 ± 0.03 | 0.82 ± 0.01 | 0.78 ± 0.03 | 0.82 ± 0.01 | 0.76 ± 0.06 | 0.13 ± 0.00 | *** |
| **C22:2n6** | 0.05 ± 0.00 | 0.03 ± 0.00 | 0.02 ± 0.00 | 0.02 ± 0.00 | 0.03 ± 0.00 | 0.03 ± 0.01 | 0.04 ± 0.00 | 0.02 ± 0.01 | 0.02 ± 0.01 | 0.06 ± 0.05 | 0.07 ± 0.07 | 0.00 ± 0.00 | *** |
| **C20:5n3** | 0.06 ± 0.01 | 0.06 ± 0.00 | 0.02 ± 0.01 | 0.04 ± 0.00 | 0.02 ± 0.00 | 0.05 ± 0.02 | 0.04 ± 0.01 | 0.05 ± 0.01 | 0.04 ± 0.01 | 0.03 ± 0.00 | 0.02 ± 0.01 | 0.05 ± 0.00 | *** |
| **C22:6n3** | 0.11 ± 0.02 | 0.12 ± 0.01 | 0.12 ± 0.02 | 0.10 ± 0.00 | 0.03 ± 0.00 | 0.38 ± 0.07 | 0.11 ± 0.00 | 0.10 ± 0.01 | 0.05 ± 0.02 | 0.03 ± 0.01 | 0.10 ± 0.03 | 0.00 ± 0.00 | *** |
| **Ʃ PUFA n-3** | 0.54 | 0.71 | 0.44 | 0.36 | 0.4 | 0.66 | 0.8 | 0.58 | 0.3 | 0.28 | 0.31 | 0.82 | *** |
| **Ʃ PUFA n-6** | 0.91 | 1.43 | 1.45 | 2.12 | 1.43 | 2.33 | 1.7 | 1.89 | 1.2 | 1.35 | 1.24 | 0.31 | ** |
| **Ʃ PUFA** | 2.07 | 2.69 | 2.29 | 2.94 | 2.26 | 3.59 | 2.85 | 3.15 | 1.9 | 2.04 | 1.95 | 1.35 | ** |
|  | **OTE-R** | **GN-R** | **NAVY-R** | **WK-1-R** | **WK-2-R** | **MAN-R** | **MAY-R** | **CRAN-1-R** | **CRAN-2-R** | **LRK-R** | **DRK-R** | **ChickP-R** |  |
| **C18:2 Trans** | 0.15 ± 0.00 | 0.10 ± 0.00 | 0.04 ± 0.02 | 0.07 ± 0.01 | 0.07 ± 0.03 | 0.06 ± 0.04 | 0.14 ± 0.03 | 0.09 ± 0.01 | 0.03 ± 0.01 | 0.03 ± 0.00 | 0.04 ± 0.02 | 0.00 ± 0.00 | *** |
| **C18:2 Cis** | 0.63 ± 0.18 | 0.72 ± 0.04 | 0.32 ± 0.02 | 0.40 ± 0.07 | 0.31 ± 0.00 | 0.39 ± 0.14 | 0.54 ± 0.07 | 0.48 ± 0.05 | 0.33 ± 0.03 | 0.35 ± 0.01 | 0.35 ± 0.04 | 0.20 ± 0.06 | ** |
| **C18:3n6** | 0.10 ± 0.00 | 0.11 ± 0.01 | 0.11 ± 0.01 | 0.16 ± 0.01 | 0.16 ± 0.00 | 0.16 ± 0.01 | 0.17 ± 0.01 | 0.14 ± 0.01 | 0.10 ± 0.00 | 0.11 ± 0.00 | 0.12 ± 0.00 | 0.00 ± 0.00 | *** |
| **C18:3n3** | 0.14 ± 0.01 | 0.17 ± 0.00 | 0.13 ± 0.01 | 0.14 ± 0.01 | 0.20 ± 0.05 | 0.13 ± 0.02 | 0.15 ± 0.03 | 0.12 ± 0.02 | 0.11 ± 0.00 | 0.11 ± 0.01 | 0.11 ± 0.00 | 0.78 ± 0.02 | *** |
| **C20:2n6** | 0.31 ± 0.03 | 0.36 ± 0.00 | 0.42 ± 0.09 | 0.66 ± 0.27 | 0.34 ± 0.15 | 0.86 ± 0.20 | 0.37 ± 0.07 | 0.46 ± 0.28 | 0.23 ± 0.01 | 0.27 ± 0.01 | 0.26 ± 0.00 | 0.15 ± 0.01 | ** |
| **C20:3n6** | 0.01 ± 0.00 | 0.03 ± 0.02 | 0.42 ± 0.15 | 0.56 ± 0.31 | 0.18 ± 0.20 | 0.74 ± 0.05 | 0.09 ± 0.09 | 0.30 ± 0.39 | 0.05 ± 0.01 | 0.04 ± 0.02 | 0.02 ± 0.00 | 0.03 ± 0.01 | ** |
| **C20:3n3** | 0.13 ± 0.00 | 0.28 ± 0.00 | 0.21 ± 0.00 | 0.08 ± 0.00 | 0.24 ± 0.00 | 0.38 ± 0.00 | 0.15 ± 0.00 | 0.27 ± 0.00 | 0.08 ± 0.00 | 0.06 ± 0.00 | 0.07 ± 0.00 | 0.02 ± 0.00 | *** |
| **C20:4n6** | 0.48 ± 0.00 | 0.65 ± 0.01 | 0.61 ± 0.09 | 0.80 ± 0.02 | 0.96 ± 0.06 | 0.71 ± 0.06 | 0.82 ± 0.00 | 0.83 ± 0.02 | 0.80 ± 0.01 | 0.82 ± 0.06 | 0.77 ± 0.00 | 0.13 ± 0.00 | *** |
| **C22:2n6** | 0.04 ± 0.01 | 0.02 ± 0.00 | 0.02 ± 0.00 | 0.02 ± 0.01 | 0.09 ± 0.09 | 0.03 ± 0.00 | 0.03 ± 0.00 | 0.02 ± 0.00 | 0.02 ± 0.01 | 0.03 ± 0.01 | 0.02 ± 0.01 | 0.00 ± 0.00 | NS |
| **C20:5n3** | 0.05 ± 0.01 | 0.04 ± 0.00 | 0.02 ± 0.01 | 0.03 ± 0.00 | 0.03 ± 0.01 | 0.05 ± 0.00 | 0.03 ± 0.00 | 0.05 ± 0.00 | 0.03 ± 0.00 | 0.02 ± 0.01 | 0.03 ± 0.01 | 0.03 ± 0.00 | *** |
| **C22:6n3** | 0.11 ± 0.02 | 0.11 ± 0.01 | 0.06 ± 0.01 | 0.12 ± 0.00 | 0.05 ± 0.01 | 0.30 ± 0.22 | 0.10 ± 0.01 | 0.23 ± 0.00 | 0.05 ± 0.01 | 0.03 ± 0.02 | 0.03 ± 0.01 | 0.00 ± 0.00 | *** |
| **Ʃ PUFA n-3** | 0.44 | 0.6 | 0.42 | 0.38 | 0.53 | 0.44 | 0.86 | 0.68 | 0.27 | 0.22 | 0.24 | 0.82 | *** |
| **Ʃ PUFA n-6** | 0.94 | 1.17 | 1.57 | 2.2 | 1.72 | 1.48 | 2.5 | 1.75 | 1.2 | 1.28 | 1.19 | 0.31 | *** |
| **Ʃ PUFA** | 2.16 | 2.6 | 2.36 | 3.05 | 2.63 | 2.61 | 3.81 | 2.99 | 1.83 | 1.88 | 1.83 | 1.34 | ** |

*Data represent means ± standard deviation (n = 2). (Otebo: Samurai (OTE); Great Northern: Powderhorn) (GN); Navy Alpena (NA); Mayacoba: Y1802-9-1 (MAY); White Kidney: WK1601-1 (WK); Cranberry: CR1801-2-2 (CRB); Manteca: Y1608-07 (MAN); Chickpea: Sierra (ChickP); Red Hawk (RH); Bellagio (BELL); Clouseau (CLOU); ND whitetail (WT)); ΣPUFA: Sum of polyunsaturated fatty acid, ΣPUFA n-6 sum of omega 6 polyunsaturated fatty acid, ΣPUFA n-3: sum of omega 3 polyunsaturated fatty acid are reported in %. The significance level was checked between the varieties at P ≤0.05,*; P ≤ 0.01, **; P ≤ 0.001,***; and NS=nonsignificant.*

**Table S5.** Phytosterol composition in Raw and Roasted Samples of 12 Bean Varieties (g kg^-1^ flour)

|  |  | **OTE** | **GN** | **NAVY** | **WK-1** | **WK-2** | **MAN** | **MAY** | **CRAN-1** | **CRAN-2** | **LRK** | **DRK** | **ChickP** | **P-value (Treatment)** | **P-value (Cultivars)** |
| --- | --- | --- | --- | --- | --- | --- | --- | --- | --- | --- | --- | --- | --- | --- | --- |
| **Phytol** | Raw | 0.14 | 0.22 | 0.31 | 0.30 | 0.29 | 0.04 | 0.27 | 0.30 | 0.26 | 0.26 | 0.27 | 0.30 | NS | *** |
|  | Roasted | ND | 0.18 | 0.28 | 0.27 | 0.30 | 0.30 | 0.32 | 0.30 | 0.26 | 0.27 | 0.27 | 0.33 |  |  |
| **stigmasterol** | Raw | 0.55 | 0.86 | 0.93 | 1.15 | 0.76 | 0.15 | 1.11 | 0.82 | 0.74 | 0.55 | 0.55 | ND | NS | *** |
|  | Roasted | 0.84 | 0.91 | 1.05 | 0.67 | 0.80 | 1.02 | 0.87 | 0.83 | 0.70 | 0.54 | 0.75 | ND |  |  |
| **β-sitosterol** | Raw | 0.67 | 1.63 | 1.51 | 1.27 | 0.42 | 0.58 | 1.21 | 1.33 | 1.03 | 0.76 | 1.00 | 0.63 | * | *** |
|  | Roasted | 1.69 | 2.37 | 1.84 | 0.97 | 0.85 | 2.48 | 1.22 | 0.89 | 0.94 | 0.66 | 1.08 | 0.84 |  |  |
| **Fucosterol** | Raw | 0.48 | 0.26 | ND | 0.29 | 0.22 | 0.11 | 0.42 | 0.44 | 0.26 | 0.24 | 0.37 | ND | NS | *** |
|  | Roasted | 0.29 | 0.26 | ND | 0.29 | 0.10 | 1.07 | 0.41 | 0.24 | 0.30 | 0.14 | 0.31 | ND |  |  |
| **Squalene** | Raw | 0.29 | 0.21 | 0.17 | 0.20 | 0.12 | 0.10 | 0.23 | 0.21 | 0.44 | 0.11 | 0.16 | ND | NS | *** |
|  | Roasted | 0.33 | 0.28 | 0.20 | 0.13 | 0.20 | 0.48 | 0.14 | 0.10 | 0.12 | 0.21 | 0.09 | ND |  |  |

*Data represent means (n = 2). Data were analyzed by one-way ANOVA according to an analysis of variance and Tukey's multiple mean comparison test (p < 0.05). (Otebo: Samurai (OTE); Great Northern: Powderhorn) (GN); Navy Alpena (NAVY); Mayacoba: Y1802-9-1 (MAY); White Kidney: WK1601-1 (WK-1); Cranberry: CR1801-2-2 (CRAN-1); Manteca: Y1608-07 (MAN); Chickpea: Sierra (ChickP); Red Hawk (DRK); Bellagio (CRAN-2); Clouseau (LRK); ND whitetail (WK-2)).* ND: Not detected; *All analyses are measured in DW (dry weight). The significance level was checked between two pairs of Raw and Roasted at ≤0.05,*; ≤ 0.01, **; ≤ 0.001,***; and NS=nonsignificant*
